# Differential experiences of discrimination among ethnoracially diverse persons experiencing mental illness and homelessness

**DOI:** 10.1186/s12888-014-0353-1

**Published:** 2014-12-14

**Authors:** Suzanne Zerger, Sarah Bacon, Simon Corneau, Anna Skosireva, Kwame McKenzie, Susan Gapka, Patricia O’Campo, Aseefa Sarang, Vicky Stergiopoulos

**Affiliations:** Centre for Research on Inner City Health, Li KaShing Knowledge Institute of St. Michael’s Hospital, 209 Victoria Street, Toronto, Ontario M5C 1N8 Canada; Université du Québec à Montréal, 405 Rue Sainte-Catherine Est, Montréal, Québec, H2L 2C4 Canada; Health Services and Health Equity Research, Centre for Addiction and Mental Health, 250 College Street, Toronto, ON M5T 1R8 Canada; Dalla Lana School of Public Health, University of Toronto, 55 College St, Toronto, ON M5T 3M7 Canada; Across Boundaries: An Ethno-racial Mental Health Centre, 51 Clarkson Ave, Toronto (Ontario), M6E 2T5 Canada; Department of Psychiatry, University of Toronto, 250 College Street, Toronto, Ontario M5T 1R8 Canada

## Abstract

**Background:**

This mixed methods study explored the characteristics of and experiences with perceived discrimination in an ethnically diverse urban sample of adults experiencing homelessness and mental illness.

**Methods:**

Data were collected in Toronto, Ontario, as part of a 4-year national randomized field trial of the Housing First treatment model. Rates of perceived discrimination were captured from survey questions regarding perceived discrimination among 231 ethnoracially diverse participants with moderate mental health needs. The qualitative component included thirty six in-depth interviews which explored how individuals who bear these multiple identities of oppression navigate stigma and discrimination, and what affects their capacity to do so.

**Results:**

Quantitative analysis revealed very high rates of perceived discrimination related to: homelessness/poverty (61.5%), race/ethnicity/skin colour (50.6%) and mental illness/substance use (43.7%). Immigrants and those who had been homeless three or more years reported higher perceived discrimination on all three domains. Analysis of qualitative interviews revealed three common themes related to navigating these experiences of discrimination among participants: 1) social distancing; 2) old and new labels/identities; and, 3) ‘homeland’ cultures.

**Conclusions:**

These study findings underscore poverty and homelessness as major sources of perceived discrimination, and expose underlying complexities in the navigation of multiple identities in responding to stigma and discrimination.

**Trial registration:**

Current Controlled Trials ISRCTN42520374. Registered 18 August 2009.

**Electronic supplementary material:**

The online version of this article (doi:10.1186/s12888-014-0353-1) contains supplementary material, which is available to authorized users.

## Background

Toronto is home to the largest cohort of homeless people in Canada, with over 5,000 people literally homeless on any given night and approximately 28,000 different individuals using homeless shelters each year [1]. Toronto is also one of the world’s most multicultural centres, with half of its population born outside of Canada, and nearly as many half (49%) describing themselves as belonging to a visible minority [2]. Newcomers represent two-thirds of the population growth in Canada over the past decade, and it is projected that by 2031, 78% of persons living in Toronto’s Census Metropolitan Area will be immigrants or Canadian-born children of immigrants [3]. Visible minorities, immigrants and refugees in Canada are especially vulnerable to becoming homeless [4-7] and homelessness, in turn, is a cause and consequence of complex health, mental health, and addiction problems [8,9].

Experiences with stigma and discrimination are distressingly common for individuals who are visible minorities, homeless, and/or have a mental illness [10-16]. Negative consequences of stigma and discrimination are well-documented, particularly negative physical and mental health outcomes, the latter at least in part due to delay or avoidance of treatment [11-13,17-27]. Increasingly, researchers have taken into account the effects of multiple domains on discrimination experiences, such as mental illness, race, gender, age, physical disability, and sexual orientation. They have typically concluded that compound effects result in even worse health outcomes, and that all of these domains are important contributors when looking for effective interventions or, as one author put it, the “capacity of the individual to resolve” the negative impacts of discrimination [28] (see also: [15,29-34]). Yet while acknowledging that discrimination can be based on multiple domains of difference, the literature largely assumes an additive, not an intersectional, effect.

Most of these primarily US-based studies also lack multicultural samples and employ quantitative measures of discrimination prevalence. And while income and neighborhood poverty are occasionally included as potential factors affecting perceived discrimation, homelessness and immigration status are rarely considered.

In part because immigrants tend to experience housing instability in the form of residential crowding - sometimes called ‘hidden homelessness’- literature exploring their experiences with literal homelessness is scant [35,36]. However, some recent studies from Canada have assessed the physical and mental health of homeless immigrant populations [11,37-40], and a few qualitative studies have explored the experiences of homeless women, youth and families, including immigrants [41-44]. One Ontario qualitative study looked at factors affecting discrimination among homeless mothers with mental illness, and noted but did not pursue the finding that “issues newcomers faced” tended to compound the barriers for these women [42] p.691. A more recent Canadian study asserted the need for a better understanding of “how discrimination and its health effects are differentially experienced” by immigrants and refugees [45], p.1557. Authors of a review of discrimination and stigma measures related to mental illness note a gap in understanding how non-Western cultures experience mental illness stigma [46]. On the whole, much remains unknown about the relative prevalence of discrimination due to homelessness and immigration status, the factors associated with that prevalence, and especially about *how* multiple identities interact and inform experiences of stigma and discrimination.

This mixed method study takes advantage of a unique study sample with an ethnically diverse group of individuals who are experiencing homelessness and mental health problems to address some of the gaps in the literature. A key purpose of this study is to explore how individuals who bear multiple labels and identities of oppression including being homeless, an immigrant, a racialized minority, and having a mental illness, navigate stigma and discrimination – and what affects their capacity to do so. Specifically, this study addresses three research questions: 1) What is the prevalence of perceived discrimination due to homelessness/poverty, race/ethnicity, and mental health/substance use in an ethnically diverse sample of homeless people with mental illness? 2) What sociodemographic characteristics are associated with perceived discrimination? And, 3) How do multiple identity characteristics interact and inform experiences of discrimination?

## Methods

Given the lack of quantitative data on perceived discrimination based on poverty and homelessness, and our interest in qualitatively exploring the interplay between identities in multiple social locations simultaneously, we determined that a mixed methods approach would be most appropriate to address our research questions. This study uses data collected in Toronto as part of a 4-year national randomized field trial of the Housing First model for persons experiencing homelessness and mental illness. Details about this national trial - “At Home/Chez Soi” – have been published elsewhere: [47,48].

### Study sample

In Toronto, At Home/Chez Soi participants were recruited from its extensive network of organizations serving people experiencing homelessness, including shelters, drop-in centres, community health centres, hospitals and criminal justice programs. Eligible participants were 18 years or older, absolutely homeless or precariously housed^a^, had a severe mental illness with or without a co-occurring substance use problem, and were not currently receiving assertive community treatment or intensive case management. Individuals not meeting these criteria were ineligible for the study, as were those who were not a Canadian citizen, permanent resident, refugee or refugee claimant at the time of enrollment. Targeted recruitment strategies, published elsewhere [47], ensured the final sample was representative of the ethno-racial diversity of Toronto and characteristic of its homeless population. From the total sample of 550 participants in Toronto, for the purposes of this study we examined the subsample of 231 who identified as racialized minorities and rated as “moderate need” for mental health services^b^. For the qualitative component we used purposeful sampling methods; field coordinators identified 36 individuals who were most willing and able to reflect on their experiences. All of those identified agreed to participate in an in-depth interview.

#### Sample description

Three-quarters of the qualitative subsample (n = 36) interviewed were male and just over half (55%) were younger than 40 years. Two-thirds had completed at least high school. One-third were born in Canada; the remainder were born in 19 different countries (see Table 1 footnote). Two-fifths had spent three or more years living homeless. Just over one-third were diagnosed with a psychotic disorder. These demographic characteristics were similar to the larger sample (n = 231), though the latter included slightly fewer males (66% compared to 75%) and lower educational achievement overall (55% completed high school or more, compared with 66% in the subsample) (Table 1).

**Table 1:** **Selected demographic characteristics**

	**Qualitative interview participants (N = 36)**		**Ethno-racial moderate needs participants (N = 231)**	
	**n**	***%*** *	**n**	***%*** *
Age				
<30 years	12	*33.3*	66	*28.6*
30-39 years	8	*22.2*	53	*22.9*
≥40 years	16	*44.4*	112	*48.4*
Gender				
Female	9	*25.0*	75	*32.5*
Male	27	*75.0*	152	*65.8*
Transgender	0	*0.00*	4	*1.7*
Education				
Less than high school	12	*33.3*	104	*45.0*
Completed high school	8	*22.2*	43	*18.6*
Some college/university	16	*44.4*	84	*36.4*
Country of birth				
Canada-born	12	*33.3*	65	*28.1*
Foreign-born	24**	*66.7*	166	*71.9*
Homelessness: Lifetime duration				
<3 years	20	*55.6*	123	*56.2*
≥3 years	16	*44.4*	96	*43.8*
Mental health: MINI results				
Current psychotic disorder	13	*36.1*	81	*35.1*

*Percentages are based on valid responses. **Countries of birth in this group included: Canada (12), Jamaica (3), Afghanistan (2), Somalia (2), Uganda (2), Bangladesh, Barbados, Congo, Egypt, Ethiopia, Ghana, Grenada, Guyana, Kenya, India, Iran, Mexico, Sri Lanka, Trinidad and Tobago, and Zambia (each, n = 1).

This study was approved by the Research Ethics Board (REB) at St. Michael’s Hospital, Toronto. At Home Study participants were recruited between October 2009 and June 2010; they provided written informed consent and were compensated for their participation. Separate REB approval was obtained for the qualitative interviews; participants signed a separate consent form and received additional compensation of $25 cash and transportation costs for in-person interviews conducted between November 2010 and July 2011.

### Data sources

#### Quantitative

We use findings from discrimination questions included in the baseline interviews with the sample of 231 ethnoracial moderate needs participants. Specifically, participants were asked whether they had “experienced discrimination or been treated unfairly by others in Canada” due to: ethnicity, race, skin colour, language, accent, religion, gender, sexual orientation, appearance, mental health, use of alcohol or drugs, homelessness, or poverty. We grouped participants according to whether they perceived experiencing discrimination due to (i) homelessness *or* poverty, (ii) mental health *or* use of alcohol or drugs, and (iii) race *or* ethnicity *or* skin colour. Selected demographic variables, adapted from the 2006 Census of Canada, were also collected and used to describe these two samples, and the Mini International Neuropsychiatric Interview 6.0 (MINI 6.0) was used to assess and describe mental health and substance use disorders [48].

#### Qualitative

A trained researcher conducted in-person interviews, lasting between 34 and 90 minutes, with the subsample of 36 ethnoracial moderate needs participants. The semi-structured interview protocol was designed to probe for experiences with intersecting dimensions of homelessness, mental illness, race and gender or any other possible forms of discrimination or stigma; for example, after general questions about discrimination experiences with homelessness, participants were asked to reflect whether/how their mental health status, ethnicity, or gender may have influenced those experiences. Though asked generally about being “treated unfairly,” the concepts of discrimination and stigma were not explicitly defined, as the intent was to better understand how the interviewees conceived of and described experiences associated with these identities. Though interpreters were available, all chose to conduct their interviews in English. All interviews were audio-taped and transcribed.

### Analytic strategies

#### Quantitative

SPSS 20.0 software was used for the quantitative analysis. Descriptive statistics, including frequencies and means, were used to describe both study samples. Cross-tabulations and Pearson Chi Square tests and Fisher’s Exact Test for cell sizes fewer than 4 were used to assess statistically significant differences of categorical demographic variables on perceived discrimination.

#### Qualitative

We used data analytical techniques of grounded theory to analyze transcribed interview text. Specifically, we used coding and comparative analytic inductive procedures to de-contextualize and re-contextualize individual cases and experiences [49]. This iterative process led to increasingly abstract conceptual categories which helped to expose the data and patterns within it [50]. The research analyst first conducted line-by-line open coding during a thorough read of the transcripts to identify key concepts, then progressively transformed codes into higher level categories by using a constant comparative method of analyzing and grouping similar codes into conceptual categories (“axial” coding). A constructionist stance guided the process, which meant the focus was to identify latent thematic schema, as opposed to semantic or descriptive [51]. A team of qualitative analysts compared several coded transcripts to ensure consistency at regular intervals throughout this process. NVivo 9.2 software facilitated the coding and thematic analysis process, and memo notes were used to track emerging codes. To enhance rigor, the researcher kept a journal to track and address potential assumptions or biases, and listened to audio-tapes after coding to gain contextual insight that may have been missed in the transcribed version.

## Results

The Quantitative section describes characteristics of perceived discrimination among our sample of 231 homeless, ethnoracially diverse moderate needs participants, while the Qualitative section synthesizes findings from the analysis of interviews with the subsample of 36 participants.

### Quantitative

#### Perceived discrimination findings

Perceived discrimination among study participants is very high, with 61.5% reporting perceived discrimination due to homelessness or poverty, 50.6% due to race, ethnicity, or skin colour, and 43.7% due to mental health problems or alcohol/drug use. Nearly three-quarters of the sample (73.6%) reported perceived discrimination on at least one of these domains (Table 2).

**Table 2:** **Prevalence of perceived experiences of discrimination in any setting during Prior 12-months, Toronto at Home/Chez Soi Ethno-Racial moderate needs participants**

	**(n = 231)**	
***Perceived discrimination due to…***	**n**	**%**
Homelessness or poverty	142	61.5
Ethnicity, Race, or Skin colour	117	50.6
Mental health or alcohol/drug use	101	43.7
*Any discrimination*	*170*	*73.6*

Univariate analysis showed that age, gender, education, employment status and the diagnosis of psychotic disorder were not related to any of the three domains of perceived discrimination. (See Additional file 1) Individuals who experienced discrimination in any of the three domains showed statistically significant difference in terms of their ethnic or cultural identity: more than half of those who experienced each form of discrimination identified their ethnicity as Black. Individuals who reported perceived discrimination due to homelessness or poverty and due to mental health or alcohol/drug problems had significantly lower monthly income compared to those who did not report any of these types of discrimination. Individuals born outside of Canada were more likely to report perceived discrimination compared to their Canadian-born counterparts, and this pattern was significant across all three forms of discrimination. Individuals who experienced discrimination of any of the three forms were more likely to have been homeless for three or more years (χ 2 (1, N = 170) = 8.80). We obtained similar results (not reported) by testing each form of perceived discrimination against the group which did not experience any type of discrimination.

### Qualitative

#### A note on perceived discrimination experiences

All of those interviewed described being stigmatized due to their differences, ranging from appearance (traditional ethnic dress, unkempt clothing due to living on the street, skin colour), to behaviors (typically associated with mental illness or substance use). And most had encountered discrimination, primarily when seeking jobs or housing, or interacting with law enforcement or service providers. Participants’ subgroup identities are inextricably linked as they intersect with perceptions of, and experiences with, stigma and discrimination. These individuals certainly do not perceive themselves nor experience discrimination categorically: as one woman commented, “I would get put down because not only am I brown but I’m a woman that’s out on the streets.” However, this qualitative component is focused not on documenting or describing these experiences, but rather on uncovering how their multiple interlocking identities affect these experiences. A companion article from the At Home/Chez Soi study, published elsewhere, explored the perceptions of discrimination in healthcare settings among this populaion [52].

#### Theme: Social distancing

A recurrent theme in participant narratives described social distancing, the reasons underlying it, and its impact on coping and recovery.

##### Reasons for social distancing

Interviewees described distancing themselves from family and friends because of the stigma associated with their experiences of homelessness and/or mental illness. One man said homelessness made him feel worthless: “I stopped trusting anyone. I’m isolating myself;” another discussed avoiding contact to ‘hide’ his mental illness: “I tried to be isolated from anyone, so no one can know that I’m sick.”

Nearly all of those who immigrated as adults additionally described distancing themselves from family and friends in their home country, declaring they had not informed them about their homelessness or, if diagnosed in Canada, their mental illness. They spoke about the shame of being homeless or mentally ill, of feelings of failure to meet social norms and expectations and not wanting to “disappoint” “worry” or “disturb” their family. Typical comments included: “I don’t want my mom to get depressed and worry about me;” and “I just didn’t want to have to put them through that.”

Many Canadian-born respondents also talked about social distancing, not trusting others, and not wanting to cause worry or be a burden; however, their families and friends were more apt to know about – and in some cases had played a direct role in - their pathways to homelessness and/or mental health problems. Several noted that their family members were also poor and busy trying to survive. Some avoided family members or friends who were substance abusers or triggers for their mental health problems as a means to aid in their own recovery.

Female interviewees were more apt to talk about social distancing as a survival technique. As this woman explained: “I don’t get myself involved in any situation I can’t come out of…I stay by myself.”

##### Varied effects of social distancing

Even when social distancing was not a direct result of stigma, and even when it was deemed necessary for recovery to progress, it had myriad negative effects which entrenched participants further into poverty and homelessness. For example, it cut off important sources of emotional or instrumental support, created and/or exacerbated depression or other mental health problems, and reinforced a lack of trust in others, thus hindering engagement with social services. All of them understood and felt the negative consequences; one articulated it this way: “I really kept myself isolated most of the time. So by doing so I lost contacts, I lost maybe job opportunities, maybe some encouragement which maybe I could have got from…friends.”

Among foreign-born interviewees, the profound sense of being alone reinforced their helplessness about finding a way out of their predicaments. As one man commented: “What else can you do. You are here in a foreign land. You can’t…run to your father…to your sister…to your mother to talk about it. The only person maybe is a caseworker, who gives you an appointment.” Yet while distancing and self-isolation impeded access to critical supports in Canada, it also served as a means to protect and retain such support from family/friends abroad. Many described their family as key to their survival: for example, “when I remember them…I find there is something in my life I can live for.” By not revealing their experiences of homelessness and/or mental illness to those most important to them, they were able to project a more positive self-image, prevent additional stigma, and retain emotional support. This recent immigrant explained why he distanced himself from his family: “I didn’t want to scare them, because I was their strength. My children, and my wife, they knew I was their hero.”

##### Summary

Discrimination and stigma associated with being homeless or having a mental illness, led all of these participants to distance themselves from family and friends, and experience profound loneliness and loss of critical supports. The motives and effects of social distancing, however, varied within and among subgroups: for example, women tended to talk about it more as a tool of survival than as a means of avoiding shame, and recent immigrants distanced themselves from family and friends in their homelands to protect loved ones and retain meaningful emotional supports.

#### Theme: Old and new labels

Another recurring theme was the qualitatively different ways that those interviewed described experiences with the three kinds of discrimination we asked about: race/ethnicity, mental illness, and homelessness. Being a person of colour is an “old label”: race discrimination was something participants were familiar with; they had grown up experiencing and learning to ignore or otherwise cope with – that is, racism was *nothing new*. Acquiring a diagnosis of a mental illness or becoming homeless, on the other hand, were mostly “new labels” and identities which required new – and pressing – internal negotiation to determine how it fit or did not fit with who they are, and thus how they would respond to stigma or discrimination based on them. Though this theme held for all participants, there were again differences between foreign- and Canadian-born participants.

##### Race/Ethnicity/Skin colour: “Old labels”

Asked directly about any experiences they might have with discrimination based on their colour or race, most of the immigrants in this sample said they *had not experienced it at all* since arriving in their new country. These responses were frequently tied to their perceptions of Canada and the multicultural context of Toronto, as illustrated by the following comments: “I heard from my friend ..[that] there is no discrimination here;” and, “I never got any racial discrimination that I knew of ….” These perfunctory assertions, though, belie the reality that they were simply accustomed to race discrimination in its many forms and had become inured to its inevitability. Their comments included, for example: “my upbringing was already training me to deal with racism;” “It doesn’t bother me because I grew up with it, I know how to deal with it;” and, “it used to bother me, but after you see something…repetitive after like, after five, ten years, you’re like, yeah, whatever.”

While Canadian-born participants were more apt to mention specific experiences of race discrimination, a few talked about it as becoming more insidious. As one woman put it, “it’s changing forms now…it’s something more sneaky.” Yet they too were much more apt to focus on stigma associated with mental illness or homelessness.

##### Homelessness and mental illness: New labels

Most expressed reactions to being diagnosed with a mental illness and/or becoming homeless in dramatic, life-altering terms: “I was kind of shocked”, “it was just this overwhelming feeling”; “oh, it ruined my whole life”; and, “mental health kind of just snatched me out of my life.” Several initially reacted with denial: “I didn’t believe it…I said I’m normal, I just had some stability issues”; and, “I couldn’t believe that I was sick.” Similarly, many– especially foreign-born participants – described becoming homeless as completely new and confusing: “it was totally new, totally new;” and “it was something all of a sudden and something that I’ve never heard about.”

For many respondents, becoming homeless was inextricably linked to their mental illness. One man described his diagnosis as the reason he was “put on the streets”; another noted his was “another thing I would have avoided if I wasn’t homeless…I wouldn’t have to have an assessment for my mental health.”

That they more frequently verbalized stigma and discrimination from homelessness than from mental illness may stem in part from homelessness being more visible, as they could more easily “hide” their mental illness from others while getting accustomed to treatment and others’ reactions: “not many people have asked me, so no, I haven’t been discriminated on the basis of mental illness;” and, “If I didn’t tell you that I had ADHD and bipolar, you wouldn’t have known, right?”

Asked about experiences with being stigmatized due to these “new label” identities, they primarily talked about the process of learning what these new labels had to do with who they were: “it was puzzling at first because I didn’t expect people to be acting that way;” and, “It’s not you who says that you are homeless. People - they define you as homeless.” The first time he encountered a homeless shelter, one immigrant man said: “I didn’t get used to the adjustment very well. I didn’t deal with it… I was trying to pretend that I wasn’t there.” Another commented: “It just feels like I’m walking but I don’t feel myself…I have really heavy bags to carry and I know that I exist but then I don’t feel myself…kind of half semi-conscious.”

##### Summary

Interviewees described experiences with discrimination associated with race, homelessness, and mental illness, in qualitatively different ways. Adjusting to race discrimination was not new, but navigating stigma associated with mental illness diagnoses and homelessness were new and unsettling experiences. For most, becoming homeless and/or diagnosed with a mental illness involved an overwhelming sense of failure, loneliness, and sadness, and many blamed themselves, thus internalizing the stigma they experienced from others. Participants frequently linked becoming homeless with being diagnosed with a mental illness, but found it easier to mask and avoid the stigma associated with mental illness.

#### Theme: ‘Homeland’ cultures

Another factor that influenced how these individuals processed “new label” identities - homeless and mentally ill – and associated stigmas, was how these were perceived in their culture and/or birth country. Though it tended to affect recent immigrants more strongly, especially those with families still in their homeland, it also affected Canadian-born respondents with relatives or friends who were still closely tied to their homeland and its culture.

##### Mental illness

Reactions to stigma associated with mental health diagnoses were shaped by perceptions of mental illness in the culture of their home country. The words they used to describe how mental illness was perceived (e.g. “outcasts” and “crazy” “dangerous” and “someone screaming and beating people”) can be read as a desire to contrast those perceptions with their personal experiences. One man said in his home country mental illness meant that “you’re a crazy person. I couldn’t believe I was sick…because…I’m belonging now to “crazy people.”

Those who struggled the most to come to terms with a medicalized, diagnostic understanding of their mental health symptoms and issues in Canada tended to be those from cultures with non-medicalized concepts of mental illness. For example, “they tend to think, like, you’re bewitched…and think a spell was put on you. They don’t take it like it might just be a medical thing.” Many responded to the cultural dissonance by further distancing themselves from –and even blaming - those individuals and cultural organizations which had previously been important sources of support. For example, one man who had immigrated to Canada as a boy blamed his inability to communicate with his parents about his experiences with depression (and treatment) on his cultural roots:“ They don’t understand what’s a mental illness.” Another man avoided taking medications to control symptoms because his priest suggested they came from “a Satanic source…he thought I had evil spirit inside me.” Though he took communion as advised, his condition worsened; he subsequently avoided church, which had been an important source of support for him. A young woman raised primarily in Canada said her otherwise supportive parents “kind of knew” she had “mental issues” but did not seek psychiatric help, and did not believe in medications, because, “[back home] they think of it in spiritual terms, not medical…so I think culture does play a huge role.”

##### Homelessness

Similarly, many respondents used very strong language to describe how people from their homeland/culture perceived homeless people: “like a dog that doesn’t have an owner… No home, no hope;” “people…really don’t talk or sit with that person;” “you can die in the street, just like an animal;” and, “someone who doesn’t deserve help.”

This perception clashed with their personal experiences: “[People back home would say] you came to the west – it’s a land of opportunity, so you should be able to better yourself…but it’s actually hard to make money.” Another said he had assumed homelessness was something that “happened to orphans or those with difficult family relations” – not to someone from a “decent family” like his; he therefore struggled when others would “look at you like it’s your fault that you’re homeless.”

Though negative stereotypes of homelessness were mostly consistent across all respondents, there were some distinctions by country of origin. One man from Colombia, for example, described homelessness as a symptom of an impoverished country where “a war is going on” – and people who are living on the street are treated as “people that need help.” A few interviewees from Jamaica noted the importance of family members in preventing homelessness: “in our culture you take your family in.”

##### Summary

Cultural perspectives and beliefs played an important role in how these individuals interpreted and adjusted to their newfound statuses as “mentally ill” and/or “homeless” and the associated stigma and discrimination they experienced. Many coming to terms with a medicalized understanding of mental illness and the reality of living homeless reacted to cultural dissonance by distancing themselves from their cultural origins.

## Discussion

Perceived discrimination was very common among this ethnically diverse urban sample of 231 homeless individuals with mental illness, especially perceived discrimination due to homelessness and poverty. Those who had spent three or more years without stable housing in their lifetime, and those who were foreign born, were especially likely to report discrimination due to homelessness/poverty, to mental health/substance use, and to race/ethnicity/skin color when compared with those with less experience living homeless and Canadian-born individuals.

As noted previously, literature on the prevalence of discrimination due to homelessness and poverty is scarce, especially in samples of homeless adults, so it is difficult to say how these findings compare to other samples. One study, for example, found 53% of a sample of 1,824 individuals with severe mental illness experienced some form of discrimination; this included 51.5% due to “economic circumstance” and 21.5% due to homelessness [30]. About a third of a sample of 500 Black and 419 Latino active substance users in another study reported discrimination due to poverty (38% of Black; 26% Latinos) [53]. Finally, about one-third of 523 individuals living with HIV-AIDS in temporary housing reported experiencing discrimination due to homelessness, but was specific to health-care settings [54]. While these examples suggest our finding of 61.5% experiencing perceived discrimination due to homelessness or poverty is high, the samples described above are all US-based with limited racial diversity, and are not directly comparable.

Our findings led us to explore, with a subgroup of 36 participants, factors which affected how they perceived and experienced stigma or discrimination associated with their race, homelessness, or mental illness, and to focus more closely on immigrants in our sample. We identified three overarching themes: social distancing; old and new labels; and “homeland” cultures. Addressing multiple identities allowed for an exploration of the complexity of these participants’ experiences, and helped identify the unique effects of culture and newcomer status.

All of these participants distanced themselves from friends and family due to shame and the stigma of being homeless and/or diagnosed with a mental illness. Aspects of this theme recur in the literature: social-isolation can be both a consequence and a survival mechanism for individuals experiencing homelessness [55,56], and stigma associated with mental illness is shown to undermine social connectedness [57]. In the present study, those born outside of Canada tended to consider their family ‘back home’ as essential to their survival yet were least apt to communicate with them about their struggles and despair. The physical distance, and having control over when/how communication occurred, meant that even if they could not give financial support, they could at least protect loved ones from worrying, and maintain aspects of much needed positive self image. This finding suggests the importance of understanding social distancing as a nuanced reaction which may vary for subgroups.

Study participants spoke very differently about discrimination due to race than discrimination due to homelessness and mental illness. The latter were ‘new labels’ which required them to internally negotiate what these identities meant for them, and were most disarming. The stigma of being homeless roused especially deep shame; unlike race, homelessness is situational and subject to at least some potential for change, and unlike mental illness, can be hard to hide from others. The role and impact of each of these labels/identities is discussed in the literature, but this study suggests they can have very different meanings and roles depending on social context, and should not be analyzed in isolation [58].

Finally, the perceptions of mental illness and homelessness in participants’ homeland or culture affected how they interpreted and adjusted to these new labels. Some research has tied cultural belief systems to mental health, though it remains predominantly focused on first-world countries [59]. Our study found participants from cultures that do not medicalize mental illness were less likely to seek/find empathy from family/friends both at home or within their local ethnic communities. Similarly, a recent study found in some countries which lacked mental health services there was a “lack of a perceived norm” about mental health, and this served as a barrier to service access for some first-generation U.S. immigrants [60]. The present study, though, found that discrimination experiences were especially distinct for those participants most closely tied to a culture or homeland outside of Canada, *regardless* of their immigration status or length of time residing in Canada. That is, this study suggests cultural ties play a larger role than birthplace in shaping experience of discrimination, and would be an important factor in a more systematic investigation.

Interest in examining discrimination along multiple domains is growing, yet remains limited to two or three social categories; more diverse study samples and alternative frameworks, such as intersectionality, should be applied to ensure a comprehensive understanding of how social classifications mutually “depend on each other for meaning and, thus, mutually construct one another and work to shape outcomes [61].” As illustrated with this study, the processes with which multiple identities interact in shaping how stigma and discrimination are experienced and negotiated are nuanced and complex. A description of one of the participants’ situations illustrates this: This man immigrated to Canada as a boy, sponsored by his uncle. When his uncle died from a heart attack a few years later, he “had no one to guide” him and became homeless. “When I became homeless it had an effect on my mental health …I was very depressed, I was sad all the time, I was hopeless. That’s why I became suicidal once. So they took me to the psychiatric hospital.” His depression led him to stop talking to his family and friends (“I isolate from everyone”). He felt shame from the stigma about his homelessness and believed his family - living abroad – would not understand his homelessness or his mental illness. His homelessness, his race, his immigrant status, and his depression – when combined with social distancing, discrimination, stigma and misfortune - all hindered his capacity to exit homelessness. To neglect any of his social categories/identities, or even to focus on one more than on others, would be to lose critical meaning of his experience and, therefore, an understanding about how best to support him. As one author noted, “to focus on a single dimension in the service of parsimony is a kind of false economy [61], p.179.” As importantly, shifting from a traditional additive approach – e.g. an individual with two minority statuses experiences more discrimination than someone with one – reveals the complex processes in which social categories and locations shape discrimination experiences and individuals’ reactions to them.

### Study limitations

This study’s limitations included: 1) Participants in this study all reside in Toronto, Ontario. While the uniquely multi-ethnic context made it possible to recruit the diverse sample for this study, it may have ameliorated effects of some of the racial discrimination these participants experienced. 2) Purposive sampling methodology meant restricting the sample to those willing and able to reflect on their experiences. And, although interpreters were available, all respondents opted for interviews in English [this also occurred in a qualitative study with homeless mothers [42]]. We thus did not explore discrimination due to language as distinct from race/ethnicity, although it did not arise even among study participants with limited English proficiency. 3) Experiences with discrimination and stigma were based on self-reports and perceptions, so it is not possible to verify specific examples given; however, perceived discrimination itself has been linked to detrimental outcomes [12].

## Conclusions

This study revealed very high rates of perceived discrimination among ethnoracially diverse persons experiencing homelessness and mental illness, and exposed underlying complexities in the navigation of multiple identities in responding to stigma and discrimination, particularly for immigrants and others closely tied to countries of origin.

Becoming literally homeless, and/or of being newly diagnosed with a mental illness, had a profoundly devastating impact on the lives of study participants. Future research should not only heed the enormity of these causes for discrimination, and explore key elements in service delivery that might support coping and resilience, but also strive to disentangle the complex effects these identities have on various subgroups; as this research revealed, for example, reactions traditionally seen as negative (such as social distancing) can have important positive consequences for women and for individuals with social support systems living abroad.

## Endnotes

^a^Absolutely homeless: no fixed place to stay for at least the past 7 nights with little likelihood of finding a place in the upcoming month. Precariously housed: housed in single room occupancy (SRO), rooming house, or hotel/motel as a primary residence AND in the past year have a history of 2 or more episodes of being Absolutely Homeless OR one episode of being absolutely homeless of at least 4 weeks duration in the past year.

^b^Criteria for establishing “moderate need” was based on an algorithm which considered community functioning, mental disorder diagnosis, co-morbid substance use, prior hospitalizations and incarcerations, and results from the Multnomah Community Ability Scale (MCAS). See additional detail in 48.

## Additional file

Additional file 1: Table S1. Chi-square tests comparing individuals with and without perceived experiences of discrimination during prior 12-months on selected socio-demographic characteristics and presence of psychosis, Toronto At Home/Chez Soi Ethno-Racial Moderate Needs Participants (n=231).
