# Supplementary material for: Differential experiences of discrimination among ethnoracially diverse persons experiencing mental illness and homelessness
Source: BMC Psychiatry. 2014 Dec 14;14:353. doi: 10.1186/s12888-014-0353-1 (PMC4275956; doi:10.1186/s12888-014-0353-1)
Supplement: Additional file 1: Table S1. — Chi-square tests comparing individuals with and without perceived experiences of discrimination during prior 12-months on selected socio-demographic characteristics and presence of psychosis, Toronto At Home/Chez Soi Ethno-Racial Moderate Needs Participants (n=231). [file 12888_2014_353_MOESM1_ESM.doc]

| **Additional file 1: Table S1**. Chi-square tests comparing individuals with and without perceived experiences of discrimination during prior 12-months on selected socio-demographic characteristics and presence of psychosis, Toronto At Home/Chez Soi Ethno-Racial Moderate Needs Participants (n=231) | | | | | | | | | | | | |
| --- | --- | --- | --- | --- | --- | --- | --- | --- | --- | --- | --- | --- |
|  | Discrimination due to Homelessness or Poverty  (N=142) | | | Discrimination due to Mental Health or Alcohol/Drug Use  (N=101) | | | Discrimination due to Race, Ethnicity or Skin Colour  (N=117) | | | Any of the three forms of discrimination  (N=170) | | |
| **Characteristic** | **n (%)** | **χ^2^ (df)** | **p-value** | **n (%)** | **χ^2^ (df)** | **p-value** | **n (%)** | **χ^2^ (df)** | **p-value** | **n (%)** | **χ^2^ (df)** | **p-value** |
| ***Ethnic or Cultural Identity*** |  | 14.45 (4) | **.006** |  | 11.11 (4) | **.025** |  | 15.45 (4) | **.004** |  | 9.80 (4) | **.044** |
| Black | 81 (57.0) |  |  | 56 (55.4) |  |  | 73 (62.4) |  |  | 99 (58.2) |  |  |
| Latin American | 10(7.0) |  |  | 6 (5.9) |  |  | 7 (6.0) |  |  | 11 (6.5) |  |  |
| Middle Eastern | 13 (9.2) |  |  | 6 (5.9) |  |  | 7 (6.0) |  |  | 13 (7.6) |  |  |
| Mixed | 20 (14.1) |  |  | 18 (17.8) |  |  | 17 (14.5) |  |  | 22 (12.9) |  |  |
| Asian | 18 (12.7) |  |  | 15 (14.9) |  |  | 13 (11.1) |  |  | 25 (14.7) |  |  |
| ***Age group*** |  | 5.05 (3) | .168 |  | 1.55 (3) | .672 |  | 1.88 (3) | .597 |  | .18 (3) | .981 |
| <30 years | 34 (23.9) |  |  | 30 (29.7) |  |  | 35 (29.9) |  |  | 47 (27.6) |  |  |
| 30-39 years | 31 (21.8) |  |  | 22 (21.8) |  |  | 24 (20.5) |  |  | 38 (22.4) |  |  |
| 40-49 years | 51 (35.9) |  |  | 35 (34.7) |  |  | 37 (31.6) |  |  | 54 (31.8) |  |  |
| ≥ 50 years | 26 (18.3) |  |  | 14 (13.9) |  |  | 21 (17.9) |  |  | 31 (18.2) |  |  |
| ***Gender*** |  | 3.88 (2) | .144 |  | .95 (2) | .622 |  | .80 (2) | .801 |  | 1.29 | .526 |
| Male | 96 (67.6) |  |  | 64 (63.4) |  |  | 77 (65.8) |  |  | 111 (65.3) |  |  |
| Female | 42 (29.6) |  |  | 35 (34.7) |  |  | 38 (32.5) |  |  | 55 (32.4) |  |  |
| Transgender/Transsexual | 4 (2.8) |  |  | 2 (2.0) |  |  | 2 (1.7) |  |  | 4 (2.4) |  |  |
| ***Country of Birth*** |  | 4.32 (1) | **.038** |  | 15.75 (1) | **.000** |  | 13.29 (1) | **.000** |  | 5.39 (1) | **.020** |
| Canadian-born | 47 (33.1) |  |  | 42 (41.6) |  |  | 46 (39.3) |  |  | 57 (33.5) |  |  |
| Foreign-born | 95 (66.9) |  |  | 59 (58.4) |  |  | 71 (60.7) |  |  | 113 (66.5) |  |  |
| ***Education*** |  | 3.95 (2) | .139 |  | 1.48 (2) | .477 |  | 2.46 (2) | .292 |  | 3.79 (2) | .150 |
| < High School | 69 (48.6) |  |  | 50 (49.5) |  |  | 56 (47.9) |  |  | 79 (46.5) |  |  |
| Finished High School | 28 (19.7) |  |  | 18 (17.8) |  |  | 23 (19.7) |  |  | 35 (20.6) |  |  |
| Some college/university | 45 (31.7) |  |  | 33 (32.7) |  |  | 38 (32.5) |  |  | 56 (32.9) |  |  |
| ***Employment status*** |  | .39 (1) | .750 |  | 1.22 (1) | .269 |  | 1.16 (1) | .358 |  | .12 (1) | .537 |
| Employed | 8 (5.6) |  |  | 7 (6.9) |  |  | 8 (6.8) |  |  | 9 (5.3) |  |  |
| Unemployed/out of labor force | 134 (94.4) |  |  | 94 (93.1) |  |  | 109 (93.2) |  |  | 161 (94.7) |  |  |
| ***Monthly income*** |  | 8.07 (2) | **.018** |  | 9.99 (2) | **.007** |  | 4.72 (2) | .094 |  | 6.33 (2) | **.042** |
| < $500 | 49 (34.5) |  |  | 35 (34.7) |  |  | 42 (35.9) |  |  | 65 (38.2) |  |  |
| $500-$999 | 66 (46.5) |  |  | 40 (39.6) |  |  | 51 (43.6) |  |  | 74 (43.5) |  |  |
| ≥ $1,000 | 27 (19.0) |  |  | 26 (25.7) |  |  | 24 (20.5) |  |  | 31 (18.2) |  |  |
| ***Lifetime duration of homelessness*** |  | 15.66 (1) | **<.001** |  | 13.76 (1) | **<.001** |  | 10.54 (1) | **.001** |  | 8.80 (1) | **.003** |
| < 3 years | 61 (43.9) |  |  | 42 (42.0) |  |  | 49 (42.2) |  |  | 80 (47.9) |  |  |
| ≥ 3 years | 78 (56.1) |  |  | 58 (58.0) |  |  | 67 (57.8) |  |  | 87 (52.1) |  |  |
| ***Presence of psychotic disorder*** |  | .91 (1) | .339 |  | .15 (1) | .698 |  | 1.46 (1) | .226 |  | .36 (1) | .547 |
| Yes | 46 (32.4) |  |  | 35 (34.7) |  |  | 37 (31.6) |  |  | 58 (34.1) |  |  |
| No | 96 (67.6) |  |  | 66 (65.3) |  |  | 80 (68.4) |  |  | 112 (65.9) |  |  |
